# Tumefactive perivascular spaces mimicking cerebral edema in a patient with diabetic hyperglycemic hyperosmolar syndrome: a case report

**DOI:** 10.1186/1752-1947-7-51

**Published:** 2013-02-21

**Authors:** Seby John, Susan Samuel, Shaheen E Lakhan

**Affiliations:** 1 Neurological Institute, Cleveland Clinic, 9500 Euclid Avenue, S100A, 44195, Cleveland, OH, USA; 2 Global Neuroscience Initiative Foundation, 9776 Peavine Drive, 90210, Beverly Hills, CA, USA

**Keywords:** Tumefactive perivascular spaces, Cerebral edema, Hyperglycemic hyperosmolar syndrome

## Abstract

**Introduction:**

Acute cerebral edema is a significant cause of death in patients treated for diabetic ketoacidosis and hyperglycemic hyperosmolar syndrome.

**Case presentation:**

We present the case of a 44-year-old African American woman admitted with acute severe headache and diagnosed with diabetic hyperglycemic hyperosmolar syndrome. Computed tomography of the head showed diffuse leukoencephalopathy, but sparing of the cortex. We were concerned for acute cerebral edema secondary to hyperglycemic hyperosmolar syndrome. Magnetic resonance imaging of the brain showed numerous collections of cystic spaces in the white matter of both hemispheres representing tumefactive perivascular spaces. Her headache improved with correction of the hyperglycemic hyperosmolar state.

**Conclusion:**

Although the clinical presentation and head computed tomography were concerning for cerebral edema, the distinctive features on brain magnetic resonance imaging helped to clarify the diagnosis and differentiate it from other processes.

## Introduction

Acute cerebral edema is a significant cause of death in patients treated for diabetic ketoacidosis and hyperglycemic hyperosmolar syndrome (HHS) and usually occurs after initiation of treatment when biochemical variables start to improve [1]. The cause of cerebral edema remains controversial. It has been proposed that a rapid decline in blood glucose concentrations after initiation of treatment with insulin might increase the brain-to-blood glucose ratio, resulting in a fluid shift to the brain, thereby possibly causing significant cerebral edema [2,3]. However, there may be cytotoxic cerebral edema even before the initiation of treatment, which may be worsened by vasogenic edema post-treatment, leading to cerebral herniation.

## Case presentation

A 44-year-old right-handed African American woman with a history of chronic hypertension, asthma and former substance-use disorder (cocaine) presented with a 2-day history of acute-onset headache. The headache was severe, throbbing, holocranial, associated with photophobia and worsened with cough. The review of systems was negative, except for poor appetite and polyuria. On examination, she was afebrile with blood pressure of 149/89mmHg, heart rate of 65 beats/minute and blood oxygen saturation of 97% on room air. She was in obvious distress due to the headache, but her general physical examination was unremarkable. Her neurological examination was normal, except for mild restriction in complete abduction in both eyes. Fundoscopic examination was negative for papilledema.

Laboratory investigations included a complete blood count, which was normal, except for thrombocytopenia (107kg/μl). A complete metabolic profile showed blood glucose 959mg/dl, sodium 121mmol/L, potassium 5.2mmol/L, chloride 84mmol/L and an anion gap of 15 (reference range: 0 to 15mmol/L). Blood urea nitrogen and creatinine were 10 and 0.78mg/dl, respectively. Serum osmolality was 308mOsmol/kg. Arterial blood gas showed pH 7.34, carbon dioxide pressure of 35mmHg, oxygen pressure of 73mmHg and bicarbonate of 19mmol/L. Her urine was positive for ketones, and her serum β-hydroxybutyric acid level was 1.76mmol/L (reference range: 0.00 to 0.30mmol/L). The rest of the evaluation, including electrocardiogram, chest X-ray, urinalysis, urine toxicology screen and blood cultures was negative.

She was diagnosed with HHS, given the findings of plasma glucose ≥630mg/dl, minimal ketones in serum and serum bicarbonate ≥15mmol/L [4]. She was treated with intravenous insulin and fluid resuscitation. This was a new diagnosis of type 2 diabetes mellitus. To evaluate the headache, she underwent a computed tomography (CT) of the brain. The head CT revealed diffuse leukoencephalopathy involving the frontal, temporal and parietal lobes. The cortex was distinctively spared, and the deep gray matter structures were preserved. Given the clinical presentation of acute severe headache and findings of possible partial bilateral abducens nerve palsy, there was concern for cerebral edema secondary to her HHS. However, treatment for raised intracranial pressure was not started, given her intact sensorium and neurolo-gical examination. Close neurological follow-up was instituted, and she remained stable while her hyperglycemia was treated.

Her condition was further analyzed by performing magnetic resonance imaging (MRI) of the brain. The brain MRI scan did not reveal an acute intracranial process, but rather white-matter abnormality throughout both hemispheres, greatest in the superior convexities and extending into the anterior temporal lobes ( 1). The findings were predominantly numerous collections of cystic spaces with smooth margins and were roughly elongated parallel to the expected direction of the vascular and radial fibers. The posterior fossa was free of dilated spaces. Many of the spaces had adjacent parenchymal hyperintensity on fluid-attenuated inversion recovery (FLAIR) and T2-weighted images. There was no abnormal enhancement after gadolinium administration. This pattern was suggestive of tumefactive perivascular spaces (PVSs). There was no evidence of osmotic myelinolysis.

**Figure 1:**
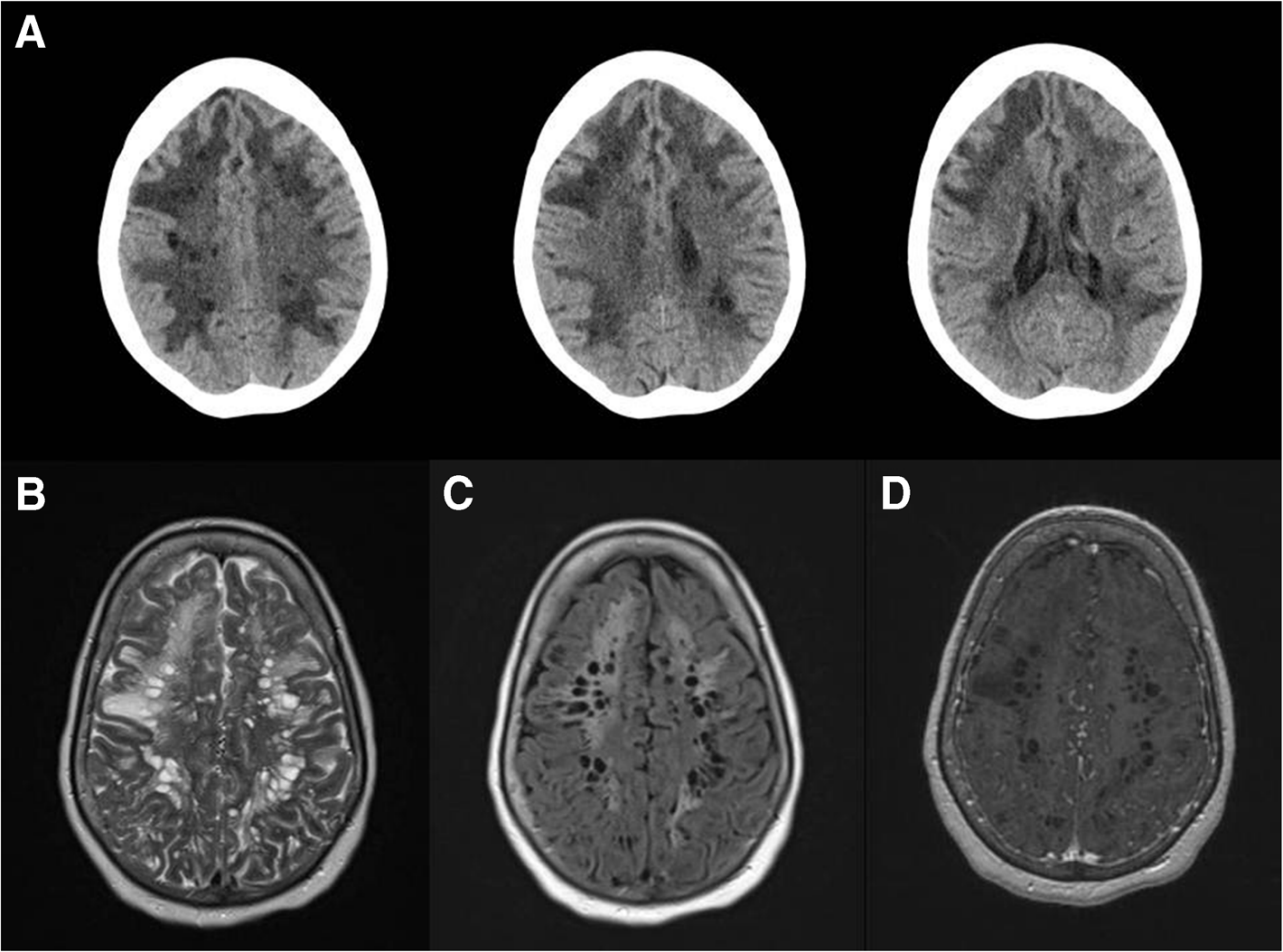
**Computed tomography and magnetic resonance imaging of the brain in the axial plane. (A)** Non-enhanced computed tomography images of the head showing diffuse leukoencephalopathy involving the frontal, parietal and temporal lobes with relative sparing of the cortex. Brain magnetic resonance imaging showed extensive white-matter abnormality throughout both hemispheres, greatest in the superior convexities, consisting of numerous collections of cystic spaces on T2-weighted **(B)** and fluid-attenuated inversion recovery **(C)** images. The spaces have smooth margins and are roughly elongated parallel to the expected direction of the vascular and radial fibers, suggestive of tumefactive perivascular spaces. Many of the spaces have adjacent T2-weighted and fluid-attenuated inversion recovery parenchymal hyperintensity. There was no enhancement after gadolinium contrast administration **(D)**.

The patient's headache improved with correction of the HHS. No further neuroimaging was performed. At the time of discharge, there were no neurological deficits evident upon examination.

## Discussion

The patient’s signs (mild partial bilateral abducens palsy) and symptoms (severe headache) resolved with correction of HHS. Such eye findings have not been reported previously in HHS; however, they were subtle in our patient. There was no evidence of raised intracranial pressure, cerebral edema, or brainstem disease or PVS. As no PVS-targeted treatment was initiated and signs and symptoms improved with HHS therapy, the patient's PVSs were deemed to be asymptomatic.

In our patient, the hypodensities on head CT were restricted to the white matter and did not correlate with the clinical presentation, thus prompting the need for MRI, which revealed tumefactive PVS. PVS or Virchow-Robin spaces are pia-lined, interstitial fluid-filled cystic structures that accompany arteries and arterioles as they penetrate the brain matter [5]. The exact etiology of PVS remains uncertain. Theories about its etiology include impaired interstitial fluid drainage secondary to lymphatic obstruction [6] or increased intraventricular cerebrospinal fluid pressure [7], altered permeability of the arterial wall as in vasculitis [8], and spiral elongation of penetrating blood vessels [9]. The development of PVS in our patient may have been a manifestation of small vessel disease [10], given her chronic hypertension, diabetes and former cocaine use.

Sometimes the PVSs become markedly enlarged and are called giant tumefactive PVSs. PVS are well-delineated on MRI. They are round or oval cystic lesions with smooth margins that are isointense with the cerebrospinal fluid and show no enhancement with contrast administration [11]. On the basis of a retrospective review of 37 cases, giant PVSs were most commonly found in the mesencephalothalamic region (21 of 37, of which nine had obstructive hydrocephalus requiring vesticulostomy and/or shunting for decompression) [11]. The most common presentation is headache followed by dizziness, dementia, visual changes, cranial neuropathy, seizure, syncope, stroke, memory problems and poor balance and concentration. When PVSs are large, they can produce surrounding parenchymal hyperintensity on FLAIR and T2-weighted imaging studies.

## Conclusions

We present an interesting case of acute severe headache in a patient with diabetic HHS whose head CT showed diffuse leukoencephalopathy that was concerning for cerebral edema. However, brain MRI demonstrated tumefactive PVS. Distinctive features discovered on MRI scans helped us to clarify the diagnosis and differentiate it from other pathologic processes.

## Consent

Written informed consent was obtained from the patient for publication of this case report and any accompanying images. A copy of the written consent is available for review by the Editor-in-Chief of this journal.

## Abbreviations

CT: Computed tomography; FLAIR: Fluid-attenuated inversion recovery; HHS: Hyperglycemic hyperosmolar syndrome; MRI: Magnetic resonance imaging; PVS: Perivascular space.

## Competing interests

The authors declare that they have no competing interests.

## Authors’ contributions

SJ, SS, and SEL participated in the development of the manuscript. All authors read and approved the final version.
